# Reconstructing the history of helminth prevalence in the UK

**DOI:** 10.1371/journal.pntd.0010312

**Published:** 2022-04-21

**Authors:** Hannah Ryan, Patrik G Flammer, Rebecca Nicholson, Louise Loe, Ben Reeves, Enid Allison, Christopher Guy, Inés Lopez Doriga, Tony Waldron, Don Walker, Claas Kirchhelle, Greger Larson, Adrian L Smith

**Affiliations:** 1 PalaeoBARN, Wellcome Trust Palaeogenomics & Bio-Archaeology Research Network, University of Oxford, Oxford, United Kingdom; 2 Department of Zoology, Peter Medawar Building, University of Oxford, Oxford, United Kingdom; 3 Oxford Archaeology Ltd., Janus House, Osney Mead, Oxford, United Kingdom; 4 York Archaeological Trust, York, United Kingdom; 5 Canterbury Archaeological Trust Ltd., Canterbury, United Kingdom; 6 Worcester Cathedral, Chapter Office, Worcester, United Kingdom; 7 Wessex Archaeology, Portway House, Old Sarum Park, Salisbury, United Kingdom; 8 Institute of Archaeology, University College London, Gower Street, London, United Kingdom; 9 Museum of London Archaeology (MOLA), Mortimer Wheeler House, London, United Kingdom; 10 University College Dublin, Belfield, Dublin 4, Ireland; NIH-National Institute for Research in Tuberculosis-ICER, INDIA

## Abstract

Intestinal helminth parasites (worms) have afflicted humans throughout history and their eggs are readily detected in archaeological deposits including at locations where intestinal parasites are no longer considered endemic (e.g. the UK). Parasites provide valuable archaeological insights into historical health, sanitation, hygiene, dietary and culinary practices, as well as other factors. Differences in the prevalence of helminths over time may help us understand factors that affected the rate of infection of these parasites in past populations. While communal deposits often contain relatively high numbers of parasite eggs, these cannot be used to calculate prevalence rates, which are a key epidemiological measure of infection. The prevalence of intestinal helminths was investigated through time in England, based on analysis of 464 human burials from 17 sites, dating from the Prehistoric to Industrial periods. Eggs from two faecal-oral transmitted nematodes (*Ascaris* sp. and *Trichuris* sp.) and the food-derived cestodes (*Taenia* spp. and *Diphyllobothrium latum* syn *Dibothriocephalus latus*) were identified, although only *Ascaris* was detected at a high frequency. The changing prevalence of nematode infections can be attributed to changes in effective sanitation or other factors that affect these faecal-oral transmitted parasites and the presence of cestode infections reflect dietary and culinary preferences. These results indicate that the impact of helminth infections on past populations varied over time, and that some locations witnessed a dramatic reduction in parasite prevalence during the industrial era (18^th^-19^th^ century), whereas other locations continued to experience high prevalence levels. The factors underlying these reductions and the variation in prevalence provide a key historical context for modern anthelmintic programs.

## Author summary

Parasitic worms (helminths) cause many health problems in poorer countries, particularly those in tropical and sub-tropical regions. In modern Europe these infections are very rare and mostly found in those travelling from endemic areas but this wasn’t always the case. Archaeological studies have detected eggs of parasitic helminths in numerous European sites. Key questions include how prevalent these infections were in past communities and whether (or when) these patterns changed over time? This paper addresses both of these questions using a large number of single grave samples from archaeological sites in England dating between Prehistoric and Industrial periods. Helminth infections were detected across all periods but the overall prevalence rates changed over time, being highest in the Roman and Late-Medieval periods. The Industrial period was interesting in that two of the three sites contained very few (or no) parasites whereas the third, London, contained high levels of infection. We discuss factors that may have contributed to the changing parasite landscape and how understanding these factors may influence efforts to control helminth infections in modern endemic areas.

## Introduction

The eggs of intestinal helminths have been detected in a variety of archaeological contexts including those in Europe and North America (reviewed in [1–6]) indicating that these parasites had a much wider historical distribution than the present day [7–9]. Helminth infections remain a concern in numerous countries, particularly those that are considered less economically developed (LEDC) in tropical and subtropical regions. Indeed, according to the World Health Organization (WHO) helminth infections rate amongst the top neglected tropical diseases [9].

Intestinal helminths are often identified in archaeological excavations because the eggs are environmentally resilient and remain identifiable by morphology for thousands of years (e.g. [10–18]). Helminth eggs can be identified to the genus level by light microscopy [19] and their presence indicates infection of a host with adult parasites. Since many parasites have complex life histories, the detection of eggs can be used to comment upon the human practices that contribute to the propagation of helminth infections. For example, detection of faecal-oral transmitted helminths (*e*.*g*. the nematodes *Ascaris* and *Trichuris*) indicates a failure of hygiene or sanitation practices to limit parasite transmission. In contrast, detection of eggs from some cestodes (tapeworms) in human derived deposits indicate the acquisition of infections from specific food sources such as freshwater fish (*Diphyllobothrium latum*, syn. *Dibothriocephalus latus*) or red meat (*Taenia* spp.), and in both cases indicate a lack of or insufficient cooking processes that would otherwise prevent parasitic infection.

Helminth eggs have been detected archaeologically in different types of faecal-associated material including latrines and other communal deposits (e.g. waste ditches), and samples associated with single individuals (e.g. the abdominal region of mummified or skeletal remains [11,14,17,20–27]. The detection of parasites in each type of context provides different kinds of information. For example, communal deposits often contain larger concentrations of parasite eggs but do not provide information related to the number of infected individuals. Although the detection of parasite eggs associated with individual remains is technically more challenging since fewer eggs are present, prevalence rates can be determined and assessed relative to other osteologically-derived characteristics including age and sex. To be most informative, individual-based datasets need to be sufficiently large to develop reliable estimates of prevalence.

Most intestinal helminths are no longer endemic in Europe, including the UK, although the timing and circumstances that led to their demise are not well understood. Although it is likely this disappearance was linked to improvements in sanitation during the 19th and early 20th centuries, prior to the development of modern anthelmintic drugs, empirically-based estimates of parasite prevalence would help to support or dispel this theory. Having established that prevalence rates of helminth infections in Medieval Europe were similar to those found in modern endemic regions [26] it was then important to consider how helminth prevalence may have changed over time. This temporal approach may reveal factors that influenced the rates of helminth infection in past populations, in particular those that led to a level insufficient to maintain an endemic status.

To assess helminth prevalence through time in a geographically limited region (historic England), we examined soil samples from the abdominal region of 464 individual skeletal remains for evidence of helminth eggs. The term prevalence usually refers to the proportion of infected individuals at a specific time. Since the samples in this study were from the pelvic (sacral) region of skeletal remains prevalence is reported as the number or percentage of burials positive for helminth eggs. Four helminths: *Ascaris*, *Trichuris*, *Taenia* and *D*. *latum* were detected and their prevalence varied across the sample sets. The goal of this study was to establish a foundation for understanding the epidemiology of parasite infections in a historical context. The changes in helminth prevalence over time can be linked with changes in the levels of urbanization, living conditions, sanitation and social practices of people in the UK. A historical perspective considering the factors which may have affected helminth prevalence in the past (without access to modern anthelmintics) has potential to inform efforts to control these infections in modern endemic contexts.

## Materials and methods

### Sample material

**Fig 1:**
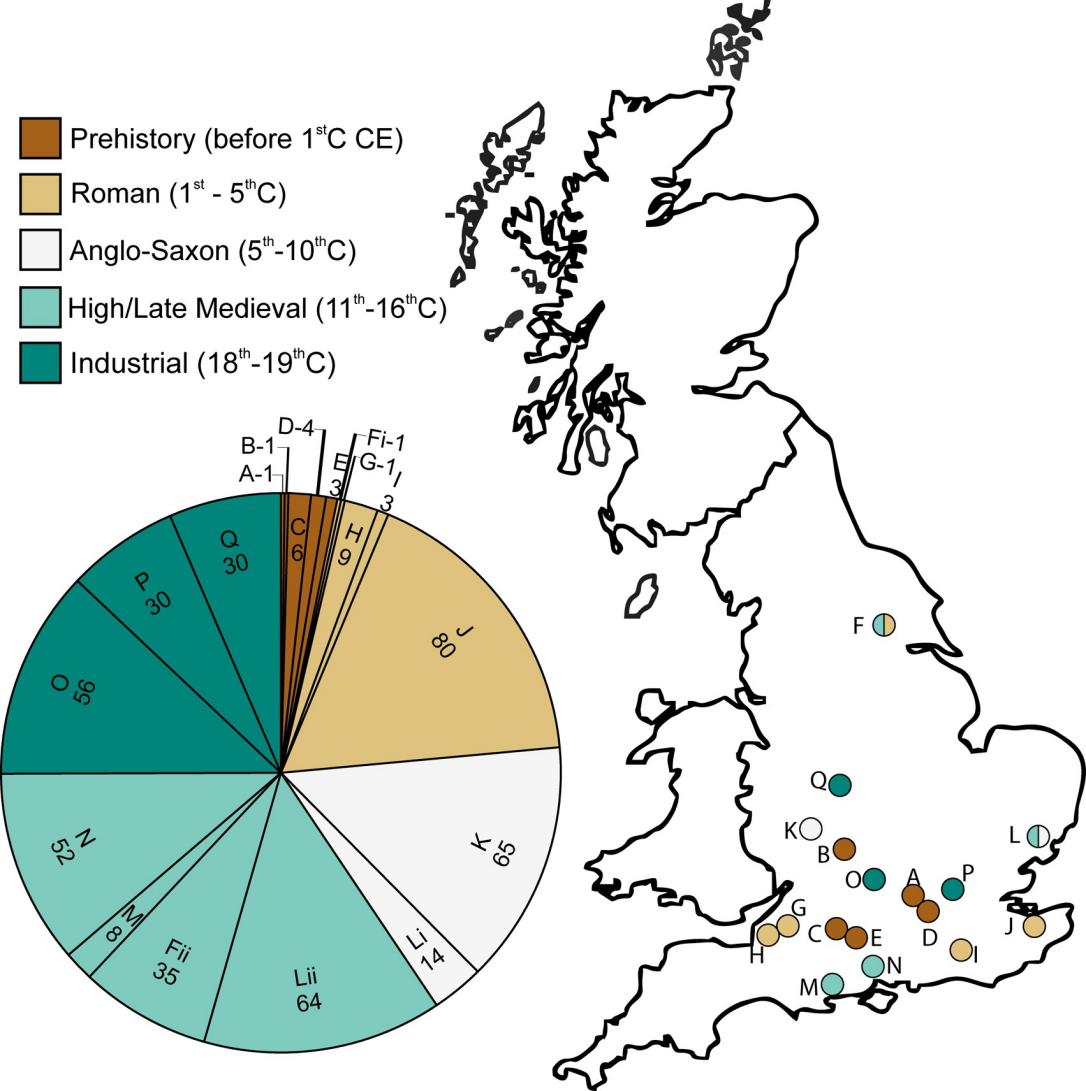
Temporal and spatial distribution of the samples. The pie chart shows the number of samples, the proportion of the sample set and the time period for each of the 17 sites of the study. The groups of samples were collected from sites depicted on the map: Taplow (Maidenhead, A), Vale Crematorium (Evesham, B), Larkhill (Durrington, C), Datchet (Slough, D), Bulford (Salisbury, E), All Saints in the Marsh (York, Roman Fi and High-Late Medieval Fii), Southern Strategic Support Main (Churchill, G), Wentwood Drive (Bleadon, H), North Park Quarry (Bletchingley, I), Peugeot Garage (Canterbury, J), Worcester Cathedral (Worcester, K), Stoke Quay (Ipswich, Anglo-Saxon Li and High-Late Medieval, Lii), Christchurch Priory (Christchurch, M), Chapel Riverside (Southampton, N), Radcliffe Infirmary (Oxford, O), St James (London, P), and Park Street (Birmingham, Q). The map was modified from the NASA SEDAC centre resource: https://sedac.ciesin.columbia.edu/maps/gallery/search?contains=United%20Kingdom.

Samples were obtained from 17 sites (Fig 1 and Table 1) which were grouped into five time periods. The time periods are: Prehistoric (Bronze Age and earlier), Roman Britain (1^st^ c BCE - 5^th^ c CE), Anglo-Saxon/Early Medieval (5^th^- early 11^th^ c CE), High/Late Medieval (11^th^-16^th^ c CE) and Industrial Era (18-19^th^ c CE). The sites include Taplow (Prehistoric, 1 sample), Evesham Vale Crematorium (Prehistoric, 1 sample), Durrington Larkhill (Prehistoric, 6 samples), Datchet (Prehistoric, 4 samples), Bulford (Prehistoric, 3 samples), York All Saints in the Marsh (Roman, 1 sample; High/Late Medieval, 35 samples), Churchill South Strategic Support Mains (Roman, 1 sample), Bleadon Wentwood Drive (Roman, 9 samples), Bletchingley North Park Quarry (Roman, 3 samples), Canterbury Peugeot Garage (Roman, 80 samples), Worcester Cathedral (Anglo-Saxon/Early Medieval, 65 samples), Ipswich Stoke Quay (Anglo-Saxon/Early Medieval, 14 samples; High/Late Medieval, 64 samples), Christchurch Priory (High/Late Medieval, 8 samples), Southampton Chapel Riverside (High/Late Medieval, 52 samples), Oxford Radcliffe Infirmary (Industrial, 56 samples), London St James (Industrial, 30 samples), and Birmingham Park Street (Industrial, 30 samples). Two of the sites, Ipswich Stoke Quay and York All Saints in the Marsh, had skeletal remains spanning two different time periods and samples from different time periods were grouped separately resulting in 19 skeletal groups for analysis. Sediment samples were collected from the pelvic region (immediately ventral to the sacrum) of the skeleton during excavation, except York All Saints in the Marsh where samples were taken from sediment adhering to the pelvis/sacrum of excavated skeletons during the cleaning process. Some of the data from York, Ipswich and Worcester has been presented in the context of prevalence rates in Medieval Europe [26]. Although “control” (e.g. sediment from the skull) were not analysed for all graves this was performed for a subset of samples; in all cases these were negative for parasite eggs. The remaining unprocessed material is archived at the Department of Zoology, University of Oxford and/or by the site archaeologist organisations.

**Table 1:** Key information on the sampled sites and the numbers positive for helminth eggs.

							Number of helminth positive samples			
Map ID^1^	Site location	Total^2^	♀_n_^3^	♂_n_^3^	Period	Dating^4^	Ascaris	Trichuris	Taenia	D. latum
A B C D E	Taplow Evesham Durrington Datchet Bulford	1 1 6 4 3	1 0 1 0 0	0 0 1 0 0	Prehistoric Prehistoric Prehistoric Prehistoric Prehistoric	Prehistoric Prehistoric LN-BA^5^ LN-BA^5^ Bronze Age	0 0 0 0 1	0 0 0 0 0	0 0 0 0 1	0 0 0 0 0
Fi G H I J	York Churchill Bleadon Bletchingley Canterbury	1 1 9 3 80	0 0 0 0 15	0 0 0 0 28	Roman Roman Roman Roman Roman	c49-45 BCE Roman Roman Roman 2^nd^–4^th^ c CE	1 1 6 0 28	0 0 0 0 0	0 1 0 0 3	0 0 0 0 1
K Li	Worcester Ipswich	65 14	14 7	26 4	Anglo-Saxon Early Medieval Anglo-Saxon Early Medieval	680-1066 9^th^-10^th^ c CE	6 5	1 1	0 2	0 1
Fii Lii M N	York Ipswich Christchurch Southampton	35 64 8 52	0 30 0 19	0 28 8 15	High/Late Medieval High/Late Medieval High/Late Medieval High/Late Medieval	11^th^-16^th^ c CE 11^th^-15^th^ c CE 11^th^-16^th^ c CE 13^th^-16^th^ c CE	13 15 0 20	3 4 0 0	0 4 0 1	0 4 0 0
O P Q	Oxford London Birmingham	56 30 30	0 0 10	0 0 11	Industrial Industrial Industrial	1770-1855 CE 1788-mid 19^th^c 1810-1873 CE	0 8 0	0 0 1	0 1 0	0 2 0

^1^Map Identifier used on Fig 1.

^2^Total numbers examined.

^3^ Number of individuals with osteological definition of sex.

^4^Provided by the site archaeologists.

^5^LN-BA: late Neolithic to Early Bronze Age.

### Processing and microscopy

In our standard approach 5-7g of the pelvic/sacral soil sample was rehydrated in 20 ml ultrapure water with gentle agitation (Titer-Tek plate shaker, setting 3/10; Titertek-Berthold, Pforzheim, Germany) over-night to disaggregate. The samples were then filtered (disposable nylon sieves, 1030 μm and 500 μm; Plastok Associates Ltd, Birkenhead, UK) and centrifuged at 400g for 5min (Heraeus Multifuge X3R). The supernatant was discarded and the pellet re-suspended in 20ml MilliQ-water and a 1ml aliquot removed for microscopy. Two subsamples (total volume 50μl) were examined for parasite eggs (Nikon Eclipse E400 with Nikon 10x/0.25 Ph1 DL and 40x/0.65 Ph2 DL objectives and QImaging MP5.0 RTV camera). Any putative eggs were recorded and images were assessed against reference images from archaeological and/or clinical contexts. Although numbers were recorded (detection limit ~80 eggs/gram of original sample) we only report the samples as positive or negative as many taphonomic factors and the size of collected sample may influence enumeration.

### Statistics

Wilson Score intervals (95% confidence level [28] and Fisher’s Exact Test were calculated using R within RStudio [29,30].

## Results

### Estimation of helminth prevalence rates in historic England

**Fig 2:**
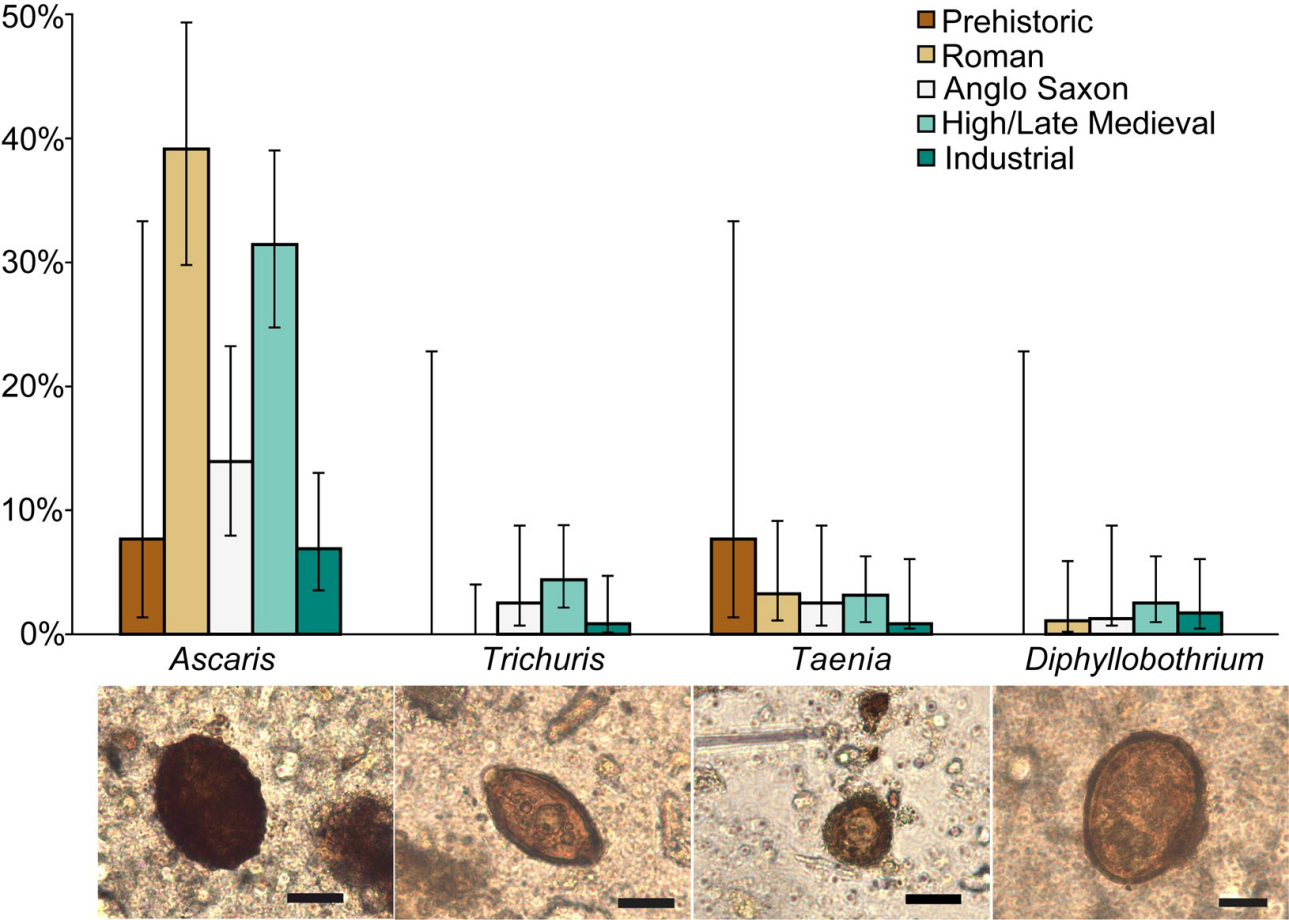
Prevalence rates of helminths through time. Prevalence of *Ascaris*, *Trichuris*, *Taenia* and *Diphyllobothrium latum* for four different time periods (Roman [n = 94], Anglo-Saxon [n = 79], High/Late Medieval [n = 159], and Industrial era [n = 116]). The confidence intervals are calculated using the Wilson Score interval (95% confidence level). Example photomicrographs of parasite eggs from left to right: *Ascaris*, *Trichuris*, *Taenia* and *Diphyllobothrium latum* Scale bar: 20 μm.

Accurate determination of the prevalence of infection is an important epidemiological characteristic. Here, we present primary data on a large dataset of single grave samples derived from different historic periods and locations within England (Fig 1 and Table 1). Overall, 134 of the 464 samples contained helminth eggs and of the 19 skeletal groups in this study, 12 showed evidence of helminth infections (Table 1). The nematodes *Ascaris* and *Trichuris* were detected in 12 groups, whilst the cestodes *Taenia* spp. and *D*. *latum* were detected in six groups (example photomicrographs shown in Fig 2). Six of the seven groups with no evidence of helminths had low numbers of skeletal samples (n≤8) with the exception of the Oxford Radcliffe Infirmary Industrial period site (n = 56). Groups with less than three samples were excluded from site-specific statistical analysis to avoid small sample size effects.

The overall prevalence of soil-transmitted nematodes (in particular *Ascaris*) was greater (24.4%, range 0–38.3%) than the prevalence of cestodes (4.1%, range 0–12.5%). This pattern was also evident when samples were grouped according to four historic intervals between the Roman and Industrial periods (Fig 2, Table 1). We have reported the detection of parasites in the Prehistoric period (Table 1) but these sample sets represented very few individuals (n = 15 from five sites, two of which only contained one sample) therefore it was inappropriate to report them as site or period prevalence estimates. Of the nematodes *Ascaris* was more prevalent (22.8%, range 0–38.3%) than *Trichuris* (2.2%, range 0–7.1%) and of the cestodes, *Taenia* was more prevalent (2.6%, range 0–14.3%) than *D*. *latum* (1.7%, range 0–7.1%). *Ascaris* was detected in 9 sites, and although *Trichuris* is transmitted by a similar faecal-oral process it was only detected in 4 sites. Of these one site contained a single *Trichuris* positive sample and no *Ascaris* positive individuals (Birmingham, Industrial period, Table 1). *D*. *latum* was found in 3 sites (in Ipswich during both Anglo-Saxon and High/Late Medieval time periods) and all of these sites also contained *Taenia* eggs (Table 1).

**Fig 3:**
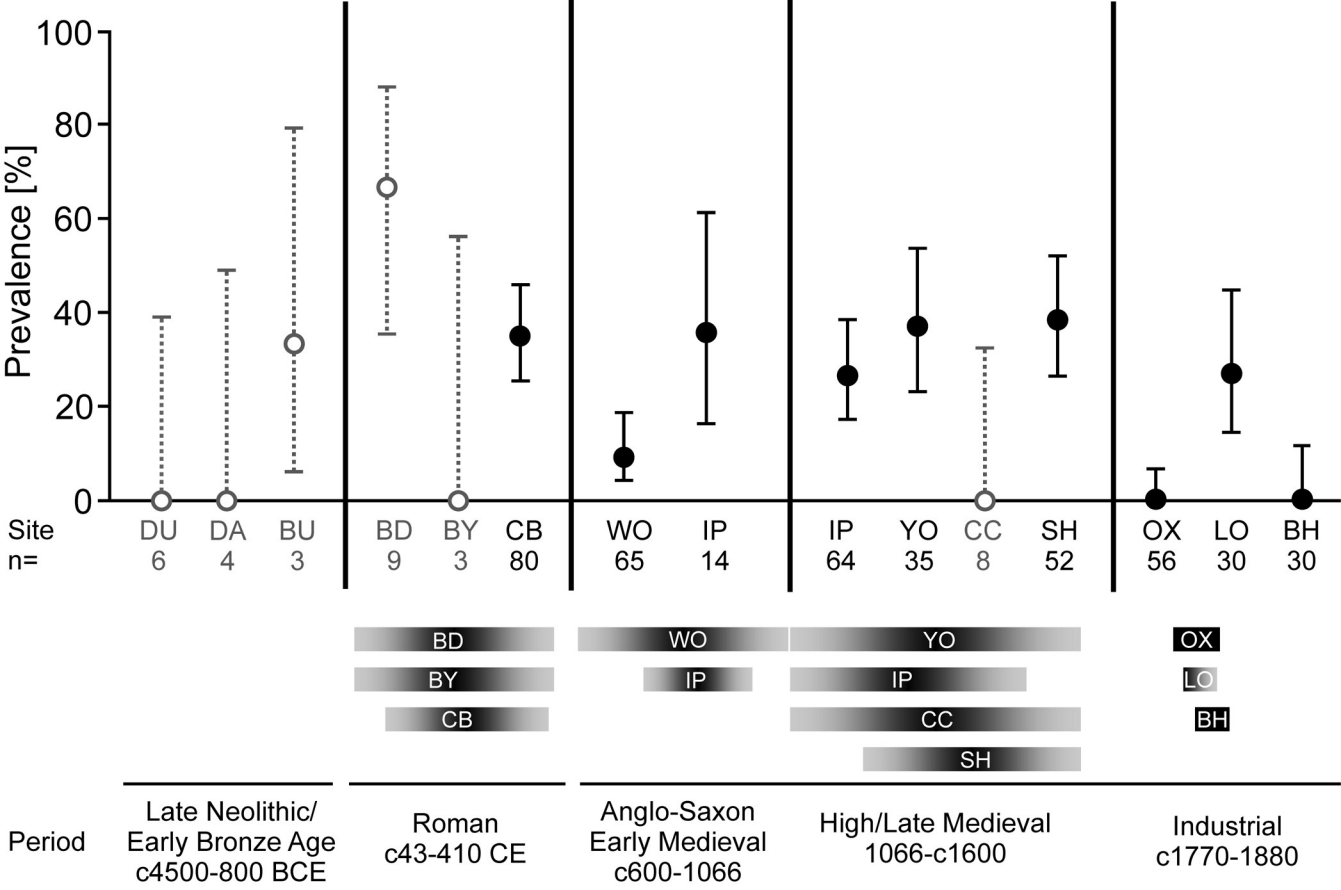
Variation of *Ascaris* prevalence rates in different sites. The prevalence of *Ascaris* in skeletal individuals from fifteen sites (group size n>1) across the five time periods is displayed with a 95% confidence interval (Wilson Confidence Interval). Sites with very few samples (<10) provide a less reliable estimate for the prevalence and are thus displayed in grey. The sites included are Durrington, DU, Datchet, DA, and Bulford, BU (Late Neolithic/Early Bronze Age); Bleadon, BD, Bletchingley, BY, and Canterbury, CB (Roman); Worcester, WO, and Ipswich (Anglo-Saxon/Early Medieval); Ipswich, IP, York, YO, Christchurch, CC, and Southampton, SH (High/Late Medieval); Oxford, OX, London, LO, and Birmingham, BH (Industrial). The dating range for the sites is visualized by the horizontal bar chart below, with a continuous time axis from the Anglo-Saxon (Early Medieval) to the Industrial periods. The dates for the earliest samples are not displayed as they are labelled Late Neolithic/Early Bronze Age (DU/DA) or Bronze Age (BU).

Since *Ascaris* was the most frequent parasite in all time periods, it represented the most robust dataset to explore changes in prevalence over time (Fig 3 and Table 1). In this analysis, the prevalence rates were generated by grouping all individuals analysed in sites associated with particular time periods and not subdivided according to location. The prevalence rates for *Ascaris* were highest in the Roman (36/94, 38.3%) and High/Late Medieval periods (50/159, 31.5%). These rates were significantly greater than those detected in the Anglo-Saxon (11/79, 13.9%; Fisher Exact p-values 0.0003 and 0.004, respectively) and the Industrial periods (8/116, 6.9%; Fisher Exact p-values both <0.0001). Although not depicted in the figure due to the relatively low numbers of graves examined, the prevalence for *Ascaris* in the Prehistoric period (1/15, 6.7%), was lower than for the Roman period (Fisher Exact p-value 0.030; Fig 3 and Table 1).

Considerable variation in prevalence rates was observed for infection with *Ascaris* between sites within each of the time periods (Fig 3). Sites with a high number of samples were considered more likely to be representative of prevalence rates in the living population. Groups with n<10 (depicted in grey in Fig 3) showed both the highest (Bleadon, 6/9, 66.7%) and lowest prevalence rates (Durrington 0/3, Datchet 0/4, Bletchingley 0/3 and Christchurch 0/8). However, of the sites with low numbers of individuals, only Christchurch was significantly different in prevalence rate to one of the other sites from the same High/Late Medieval period (Southampton, Fisher Exact p-value 0.043).

The only sites with n>10 where *Ascaris* was not detected were dated to the Industrial period (Oxford, n = 56 and Birmingham, n = 30), and there was a significant difference between these and the third site from the same period (London, 8/30, 26.7%, Fisher Exact p-values <0.005 for both sites). The prevalence rates at the two Anglo-Saxon sites were also significantly different (Fisher Exact p-value 0.021), and the site with the larger sample size (Worcester, n = 65) had the lower prevalence rate. When comparing sites with group sizes n>10, it was apparent that there was no significant difference between the Roman and the Medieval sites. These prevalence rates are aligned with the sites with the highest prevalence rates from the Anglo-Saxon and Industrial periods but the average prevalence rates in these periods were significantly lower than that of the Roman and Medieval periods.

**Fig 4:**
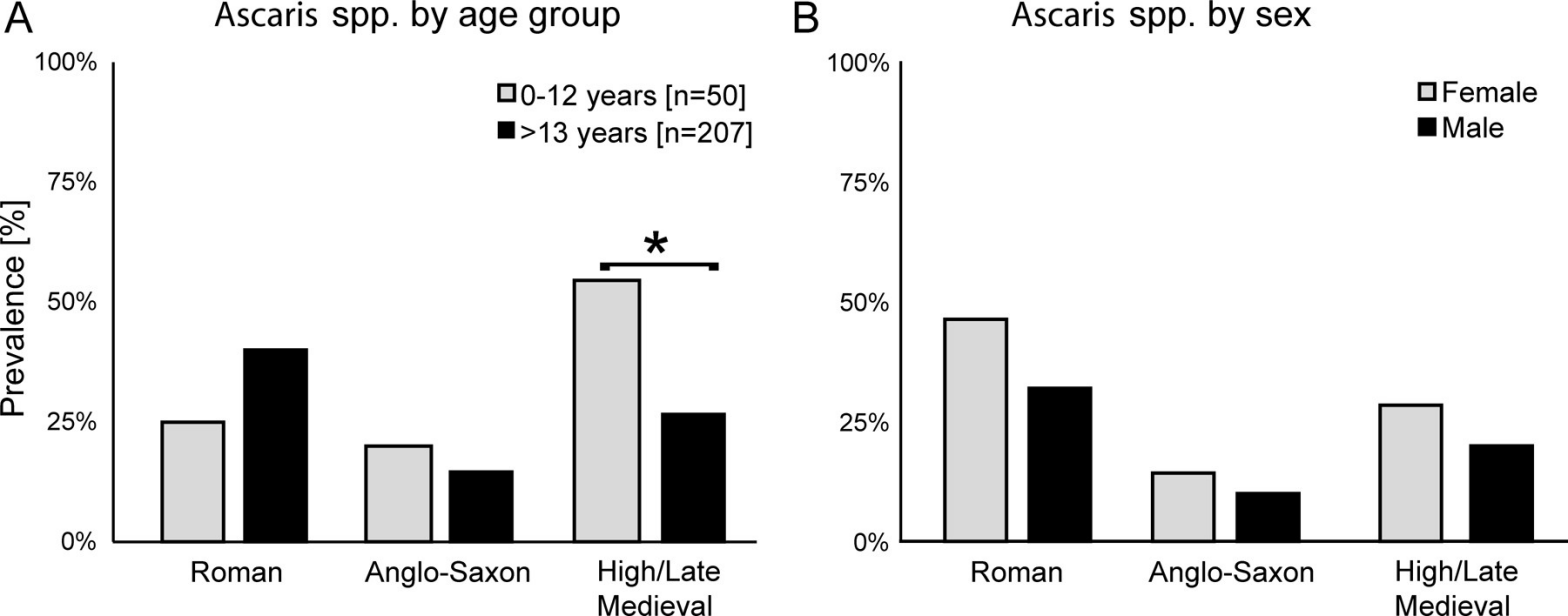
Age and Sex-associated effects on prevalence rates for *Ascaris*. Samples with associated osteological age were grouped into juveniles (0–12 years old, n = 50) and adults (>13 years old, n = 207) and according to period (A). The prevalence in juveniles was significantly higher in the High/Late Medieval period (Fisher Exact two-tailed p-value 0.02). Individuals where osteological sex was identified (adult individuals only, n = 194 with n_female_ = 85 and n_male_ = 109) were grouped by period (B). No significant differences were identified.

Estimated osteological age was available for 265 skeletal individuals. Age estimation was based on standard osteological markers [31]. Infections with soil-transmitted helminths (STH) are often more prevalent in children of school age (e.g. [32–34]), To explore this potential effect whilst maintaining sufficient group sizes for statistical analysis the data was partitioned into two groups: according to reported skeletal age: adults (>13 years old) and juveniles (<13 years old). Overall, there was a greater number of adults (n = 215) than juveniles (n = 50) in the overall dataset. To avoid any bias introduced by low sample numbers for a particular location or previously noted changes in prevalence rates over time the samples were analysed within each time period (Fig 4A). A higher prevalence of *Ascaris* was detected in juveniles (compared with adults) from the High/Late Medieval period (n = 124, 12/22 infected juveniles vs. 25/102 infected adults, Fisher-Exact p = 0.009), but not for the Roman (n = 73, 2/8 infected juveniles vs. 26/65 infected adults, Fisher-Exact p = 0.70) or Anglo-Saxon (n = 68, 4/20 infected juveniles vs. 7/48 infected adults, Fisher-Exact p = 0.72) periods. The two cestodes *Taenia* and *D*. *latum* were detected at low prevalence rates however, cestode eggs were only detected in older individuals (>13 years old).

Osteological derived identification of sex was available for 218 individuals with a slight bias towards males (n = 121) compared to females (n = 97). None of the individuals with sex information from the Prehistoric (2 females, 1 male) or Industrial periods (10 females, 11 males) were infected by *Ascaris*. There were no significant differences in infection rates between male and female individuals within any time period (Roman: 7/15 females vs. 9/28 males, Fisher-Exact p = 0.51; Anglo-Saxon: 3/21 females vs. 3/30 males, Fisher-Exact p = 0.68; High/Late Medieval: 14/49 females vs. 10/51 males, Fisher-Exact p = 0.35) (Fig 4B).

The prevalence rates of *Trichuris* in the seven sites analysed here ranged from 0% to 8.6%. The highest prevalence rate detected in this study is lower than the rate recently reported from Medieval European sites [26], where the prevalence of *Trichuris* was 28.6%. The total number of individuals in this study from Early to Late Medieval sites in which *Trichuris* was identified (9/208) was significantly lower (Fisher-Exact p = 0.0022) than the numbers reported in non-English sites (Pohansko, Brno, Ellwangen and Rottenburg) that date to the same time period (41/406). This difference, however, was due to the inclusion of the Czech site of Pohansko, which contained a high proportion of juveniles which are more likely to contain eggs of *Trichuris* [26]. When the English and European [26] data sets were segregated into juveniles and adults there were no significant differences in the Medieval prevalence rates of *Trichuris*. An alternative way of comparing *Trichuris* between sites whilst correcting for local factors that might affect prevalence for all faecal-oral transmitted nematodes would be to consider each species as a fraction of total STH detected. The prevalence of *Trichuris* as a fraction of total STH prevalence was not different between any of the Medieval English sites and those in continental Europe (Pohansko, Brno, Ellwangen and Rottenburg; primary data from [26]). It is noteworthy that very few *Trichuris* positive individuals were detected in English sites outside of the Medieval era with no *Trichuris* eggs detected in the Roman period or earlier (109 individuals) and only a single individual in the Industrial period (116 individuals).

## Discussion

In the past, many intestinal helminths were geographically more widely distributed and endemic in Europe and North America (e.g. [1–4,26,35–40]), but they are now largely restricted to tropical and sub-tropical regions [9]. Investigating the epidemiology of helminth parasites in historical contexts helps us understand the living conditions, diet, health and hygiene practices, as well as providing insights into the biology and control of infectious disease in the past (e.g.1-6.41–43). The reductions in helminth infections in Europe occurred prior to the development of modern anthelminthic drugs and identifying factors that influenced helminth epidemiology in historic populations may provide information relevant to modern control regimens.

Most archaeological studies of helminths report eggs within communal deposits, such as latrines, pit fills or waste deposits. The density of parasite eggs in communal deposits serves as an indicator of the overall amount of infection in a locality, although the number of contributing individuals cannot be determined. Analyses based on single individuals, such as mummified bodies, burial-derived sediments or coprolites, are less common. Unfortunately, most individual-based studies involve very few individuals and therefore reliable prevalence rates cannot be estimated. In the most extensive study of historic prevalence, the numbers of infected individuals in Medieval Europe was shown to be similar to modern day endemic regions [26], indicating that later reductions in local transmission were not facilitated by low initial prevalence rates. The current study builds upon these findings by determining helminth prevalence at sites within England from Prehistoric to Industrial periods. It is important to note that the prevalence rates based upon detection of eggs in pelvic/sacral soil may represent an underestimate due to taphonomic processes or sample processing (reviewed in [39–44]). The current study employed a simple water-based rehydration/filtration/microscopy approach and identified the eggs of four helminths: *Ascaris*, *Trichuris*, *Taenia* and *Diphyllobothrium latum*. All of these parasites have been reported in archaeological sites within the UK, but previous data sets were unsuitable for calculating prevalence either by being based upon communal deposits or low numbers of individuals. While burial-derived skeletal remains may not represent a totally unbiased sample of the living population (e.g. age/sex based mortality bias, local customs or taphonomic processes) these factors can be taken into account. Since school age children are more often affected by STH in modern endemic areas [32–34] in some analyses we considered adults and juveniles separately. A higher rate of infection with *Ascaris* was detected in juveniles compared with adults from the High/Late Medieval period but not in samples other periods. In contrast, eggs of food-derived cestodes (*Taenia* spp. and *D*. *latum*) were only detected in adults.

*Ascaris* was the most common infection detected throughout the whole dataset with prevalence rates greater than 20% in Roman (Bleadon, Canterbury), Anglo-Saxon (Ipswich), High/Late Medieval (Ipswich, York and Southampton) and Industrial period (London) sites. These rates are similar to those reported from late 4^th^ century Florence (27.7%) [25], Medieval Rottenburg (22%), Ellwangen (22.1%), Pohansko (35.1%) and Brno (42.9%) [26]. *Ascaris* was also detected in one of three late Neolithic/early Bronze Age sites (Bulford), but due to the small sample sizes the prevalence rates cannot be accurately estimated. *Ascaris* was detected in all Roman and Medieval period sites except for Bletchingley and Christchurch, where no parasite eggs were detected albeit in a small number of individuals (Table 1). *Ascaris* eggs were previously reported in pre-Roman English sites including Somerset [45], York [46] and Carlisle [47], as well as in a number of sites on mainland Europe (e.g. [1,10,17,38]).

*Trichuris* eggs were readily detected in Anglo-Saxon (early Medieval) and High/Late Medieval sites but were not detected in any pre-Roman (n = 15) or Roman period dated samples (n = 94) despite 37 being positive for *Ascaris* eggs. The lack of *Trichuris* in Roman and earlier samples was surprising given that *Trichuris* and *Ascaris* share a common faecal-oral pattern of infection. Although we did not detect any *Trichuris* eggs in pre-Roman sites, these parasites have previously been reported from excavations in the UK [18,45,48] and continental Europe (e.g. [1,10,38]). Interestingly, *Trichuris* has been regularly detected in Roman sites across continental Europe (summarised in [49,50]) and has been reported in the UK [41,47,51–57]. It is noteworthy that in UK sites which cover multiple time periods, *Trichuris* is more often associated with medieval deposits than those dated to earlier periods [46,51,58,59]. Hence, it is clear that *Trichuris* was present in the UK during Roman and pre-Roman periods, but may have been less common than *Ascaris*.

During the Medieval period both *Trichuris* and *Ascaris* were prevalent in English communities being found at similar levels to those in continental Europe [26]. The High/Late Medieval period represented a peak in prevalence rates in England with overall lower numbers detected in samples dated from the Anglo-Saxon/Early Medieval period (albeit with variation between sites). The eggs of both parasites are readily detected in communal medieval deposits from the UK and often found in high numbers [14,46,47,51,58–68]. Unfortunately, the data was often not quantitative or based on single samples, which makes it difficult to compare between studies. Nevertheless, it is clear that both *Ascaris* and *Trichuris* were common infections during the Medieval period. Various factors could have been associated with increased STH rates including the magnitude or patterns of trade, increased urbanisation, issues with waste disposal, sanitation or hygiene, and the use of night soil as a fertiliser [69–71]. However, with current data sets it is difficult to identify factors that adequately explain the emergence of *Trichuris* as a more common infection.

The pattern of STH prevalence rates observed in the Industrial period sites (Oxford, Birmingham and London) was different to the Medieval period. No eggs were detected in samples from Oxford (n = 56) and only one individual from Birmingham (n = 30) had *Trichuris*. In contrast, a high prevalence of *Ascaris* was evident in the samples from London although no *Trichuris* eggs were detected. There are very few reports of parasite analyses in the UK covering the Industrial period. Jones and Phipps [72] reported no helminth eggs in 12 individuals from the Spitalfields area of London whereas Anastasiou et al, [73] reported *Trichuris* (2 of 5 coprolites) and *Ascaris* (1 of 5). Akeret et al. [68] studied communal/pit fill samples from York (Low Petergate) and reported a single *Trichuris* egg in one of the Industrial period samples compared with larger numbers of *Ascaris* and *Trichuris* eggs in multiple Medieval period samples. The overall pattern of STH infections during the Industrial period suggests that prevalence rates were highly variable. The high prevalence of *Ascaris* in London clearly indicates efficient STH transmission, as might be expected from a high-density urban population with poor sanitation. The absence of helminths in the Oxford Radcliffe Infirmary burial ground samples was unexpected, but the association with a hospital that had a largely rural catchment [74] and a well-based water supply [75] may be important. Birmingham was even more surprising since the sampled site represented a densely populated urban population. However, it is noteworthy that Birmingham was also much less affected by the cholera outbreaks of the mid 1800s compared with many other towns and cities. The low cholera rates were attributed to a greater reliance on spring and well water rather than other, more easily contaminated surface water supplies [76–78] which would also affect water-based sources of STH infections. Some of the low infection rates during this period may also reflect changes in the use of night soil [70].

The prevalence of STH was variable between sites in all time periods examined which probably reflects local differences in the conditions that promote transmission (e.g. faecal contamination of food and water) or maturation/survival of eggs. Interestingly, there were significant differences in prevalence rates during different historic periods. The highest rates of STH were seen in samples from Roman and High/Late Medieval sites, with a slight decrease in the overall rate during the Anglo-Saxon/Early Medieval period. These high rates may be due to population density alongside poor separation of contaminated faecal material from the local environment, food or water sources. The slight reduction during the Anglo-Saxon/Early Medieval period may have been due to the decentralisation of people and a less urbanised society [79] reducing faecal contamination of the environment. However, the levels of STH in Anglo-Saxon sites were variable and more sites will be required to provide a clearer picture of this period. The lack of STH in two of three sites representing the Industrial period is noteworthy and may represent the beginnings of wider scale reductions in STH infection levels that led to the modern non-endemic state.

The rates of many faecal-oral infections would have been reduced by the large-scale municipal improvements to sewage and water treatment in the 1860s and the declining use of night soil to fertilise crops. The cholera outbreaks between 1831–1866, famously linked to contaminated drinking water by John Snow [80] were a key driver in sanitary reform (alongside endemic Typhoid) involving infrastructural improvement, legislation and promotion of municipal responsibility [76,81,82]. However, many interventions were piecemeal with some areas benefiting earlier than others [82]. Our data identifying very low STH levels in some locations during the Industrial period suggests that some areas may have been reaping benefits from improvements in sanitation and water supplies prior to the Victorian sanitary revolution. It is possible that our small sample (3 locations) is not representative of the Industrial period as a whole. However, given the numbers of individuals analysed and the fact that all Medieval and Roman sites with more than 10 individuals were positive suggests that this is an accurate observation. Since the enteric bacterial pathogens remained problematic during the Industrial period it is worth considering how different pathogens might be affected by variations in sanitary improvement. Helminth eggs are large compared with bacterial pathogens, which means that settlement processes (e.g. sedimentation tank or pit), used for many centuries in different guises [83], may differentially retain parasite eggs. However, the solid matter from these features needed to be periodically removed and since helminth eggs remain viable for months (e.g. [84,85]) the disposal of this material may have facilitated STH transmission. Improved settlement techniques, (e.g. holding reservoirs) and the development of sand filtration in the early 1800s [81] would have reduced contamination of water with the relatively large STH eggs. Similarly, the locations of sewage outlets and potable water intakes might differentially affect pathogens depending on sedimentation rates, water conditions and environmental resilience. Linking STH rates with local water and waste management practices during the Industrial and post-Industrial periods will be an interesting avenue for future studies.

Even though the great sanitary reforms of the Victorian period clearly impacted STH prevalence there are reports of a problem persisting into more modern times [86] Evidence for the continued, spatially focussed, problem includes STH reported in Cornish tin mine-associated populations but not similar mining communities in Staffordshire or Shropshire [87]. Significant STH infection rates (12.5%) were also reported in patients at Guys Hospital, London in 1905 [88]. Outbreaks of STH infections were reported in various mental health institutions across England in the late 1960s but there was no evidence for significant transmission of STH in the general population at this time [89].

Many factors may affect overall STH prevalence including age-structure of the sampled population ([26,32,90] and this paper) as well as a variety of site-specific factors. Therefore we considered the contribution of *Trichuris* or *Ascaris* to the overall STH burden and confirmed the observation that the level of *Trichuris* (in relation to total STH) was unexpectedly low before and after the Medieval period. In the Medieval period, *Trichuris* contributed to the STH burden at a higher level, comparable to that in Medieval samples from continental Europe. A similar approach was used to show that the parasite assemblage in North America was dominated by *Trichuris* in the 18^th^ century, but changed during the early to mid-19^th^ century when *Ascaris* became dominant [91]. STH eggs are readily detected in archaeological contexts across a wide temporal range (e.g. [10,17,18,21,38,92,93]) which argues against differential preservation as an explanation for the lack of *Trichuris* in some time periods. The changes in the proportions of the two dominant STH parasites suggest that there are factors which differentially influence transmission and these may relate to embryonation, survival or persistence of viable eggs [84,85,94].

The detection of cestode eggs (*Taenia* spp. and *D*. *latum*), albeit at lower rates than seen with *Ascaris* provides information on diet and culinary practices in English sites through the ages. Both parasites are transmitted to humans via uncooked or undercooked meat (or their products) with *Taenia* acquired from pork or beef and *D*. *latum* acquired from freshwater fish. Cestode eggs were identified in samples that spanned a wide range of time from the Bronze Age (Bulford) to the Industrial period (London). Interestingly in four of the six sites where *Taenia* was detected, *D*. *latum* was also detected suggesting local culinary preferences. To complete the life cycle the eggs from human faeces would also need to access appropriate intermediate hosts. Hence, the presence of *D*. *latum* eggs in humans indicates faecal contamination of freshwater sources of fish. In contrast, *Taenia* sp. utilise pigs or cattle as intermediate hosts necessitating exposure of livestock to human faecal material perhaps through the use of night-soil applied to pasture or animal-feed crops. Interestingly, and in contrast to STH, the cestode eggs were only detected in adults. This pattern has been reported previously [26] and may be expected from a long-lived infection acquired by occasional consumption of contaminated food.

In summary, the prevalence of helminth infections is poorly understood in past populations, despite being a key factor in the epidemiology of these infections. The findings presented indicate that the Medieval period was a high point of STH prevalence in the UK. Interestingly, outside of the Medieval period there was much less *Trichuris* present than expected which may relate to as yet undefined environmental or anthropological factors. The Industrial period saw some sites with very low levels of STH infections, but this reduction was patchy and high *Ascaris* prevalence persisted in London. Defining factors that affected the past prevalence of helminths help us understand historical patterns of disease and could be relevant to modern control programmes.

## References

[1] Bouchet F., Harter S. and Le Bailly M. The state of the art of paleoparasitological research in the Old World. *Memórias do Instituto Oswaldo Cruz*, 2003; 98: 95–101. doi: 10.1590/s0074-02762003000900015 12687768

[2] Goncalves M. L. C., Araujo A. and Ferreira L. F. Human intestinal parasites in the past: New findings and a review. Memorias Do Instituto Oswaldo Cruz, 2003; 98: 103–118. doi: 10.1590/s0074-02762003000900016 12687769

[3] Reinhard K. J., Ferreira L. F. Bouchet, Sianto F., Dutra L., Iniguez J. M. F., et al., Food, parasites, and epidemiological transitions: A broad perspective. Int J Paleopathol 2013; 3(3): 150–157. doi: 10.1016/j.ijpp.2013.05.003 29539449

[4] Araujo A., Reinhard K. and Ferreira L. F. Palaeoparasitology—Human Parasites in Ancient Material. Adv Parasitol 2015; 90: 349–387. doi: 10.1016/bs.apar.2015.03.003 26597072

[5] Flammer P.G. and Smith A.L. Intestinal helminths as a biomolecular complex in archaeological research. Phil Trans R Soc Lond B Biol Sci 2020; 375: 20190570 doi: 10.1098/rstb.2019.0570 33012232 PMC7702790

[6] Le Bailly M. Maicher C. Roche K. and Dufour B. Accessing Ancient Population Lifeways through the Study of Gastrointestinal Parasites: Paleoparasitology. Appl. Sci. 2021; 11, 4868.

[7] Pullan R. L. and Brooker S. J. The global limits and population at risk of soil-transmitted helminth infections in 2010. Parasit Vectors 2012; 5: 81. doi: 10.1186/1756-3305-5-81 22537799 PMC3419672

[8] Pullan R. L., Smith J. L., Jasrasaria R. and Brooker S. J. Global numbers of infection and disease burden of soil transmitted helminth infections in 2010. Parasit Vectors 2014; 7: 37. doi: 10.1186/1756-3305-7-37 24447578 PMC3905661

[9] WHO. (2020). *Soil-transmitted helminth infections*. Retrieved 20 April 2021, from https://www.who.int/news-room/fact-sheets/detail/soil-transmitted-helminth-infections.

[10] Aspock H., Flamm H. and Picher O. Intestinal parasites in human excrements from prehistoric salt-mines of the Hallstatt period (800–350 B.C.). Zentralbl Bakteriol Orig A 1973; 223(4): 549–558 4146831

[11] Aspock H, Auer H and Picher O. *Trichuris trichiura* eggs in the neolithic glacier mummy from the Alps. Parasitology Today 1996; 12(7): 255–256.

[12] Jones AKG. A coprolite from 6–8 Pavement. In Hall A. R., Kenward H. K., Williams D. and Grieg J. R. A. eds. Environment and Living Conditions at Two Anglo-Scandinavian Sites. The Archaeology of York, Council for British Archaeology for the York Archaeological Trust. 1983; 14 (4): 225–229.

[13] Shin D. H., Lim D. S., Choi K. J., Oh C. S., Kim M. J., Lee I. S., et al. Scanning electron microscope study of ancient parasite eggs recovered from Korean mummies of the Joseon Dynasty. J Parasitol., 2009; 95(1), 137–145. doi: 10.1645/GE-1588.1 18601576

[14] Mitchell P. D., Yeh H. Y., Appleby J. and Buckley R. The intestinal parasites of King Richard III. Lancet 2013; 382: 888. doi: 10.1016/S0140-6736(13)61757-2 24011545

[15] Le Bailly M., Landolt M. Mauchamp L. and Dufour B. Intestinal parasites in First World War German soldiers from "Kilianstollen", Carspach, France. PLoS One 2014; 9(10): e109543. doi: 10.1371/journal.pone.0109543 25333988 PMC4198135

[16] Yeh H. Y., Pluskowski A. Kalejs U. and Mitchell P. D. Intestinal parasites in a mid-14th century latrine from Riga, Latvia: fish tapeworm and the consumption of uncooked fish in the medieval eastern Baltic region. J Arch Sci 2014; 49: 83–89.

[17] Flammer P. G., Dellicour S. Preston S. G. Rieger D. Warren S. Tan C. K. W. Nicholson R. et al. Molecular archaeoparasitology identifies cultural changes in the Medieval Hanseatic trading centre of Lubeck. Proc Roy Soc B. 2018; 285: 20180991 doi: 10.1098/rspb.2018.0991 30282648 PMC6191690

[18] Ledger M. L., Grimshaw E. Fairey M. Whelton H. L. Bull I. D. Ballantyne R. et al. Intestinal parasites at the Late Bronze Age settlement of Must Farm, in the fens of East Anglia, UK (9th century B.C.E.). Parasitology: 2019; 1–12. 10.1017/S0031182019001021 31391134

[19] Bogitsh B. J., Carter C. E. and Oeltmann T. N. Human parasitology. 4th ed. Amsterdam, Boston. Elsevier, Academic Press 2012.

[20] Ruffer M. A. Note on the Presence of "Bilharzia Haematobia" in Egyptian Mummies of the Twentieth Dynasty [1250–1000 B.C.]. Br Med J 1910; 1(2557): 16. 10.1136/bmj.1.2557.16-a PMC2330583 20764829

[21] Bergman J. Stone age disease in the north—Human intestinal parasites from a Mesolithic burial in Motala, Sweden. J. Arch. Sci. 2018; 96: 26–32.

[22] Shin D. H., Oh C. S., Chai J. Y., Lee H. J. and Seo M. Enterobius vermicularis Eggs Discovered in Coprolites from a Medieval Korean Mummy. Korean J Parasitol 2011; 49(3): 323–326. doi: 10.3347/kjp.2011.49.3.323 22072838 PMC3210855

[23] Nezamabadi M., Mashkour M., Aali A., Stollner T. and Le Bailly M. Identification of Taenia sp. in a natural human mummy (third century BC) from the Chehrabad salt mine in Iran. J Parasitol 2013; 99(3): 570–572. doi: 10.1645/12-113.1 23240712

[24] Soe M. J., Nejsum P., Fredensborg B. L. and Kapel C. M. DNA typing of ancient parasite eggs from environmental samples identifies human and animal worm infections in Viking-age settlement. J Parasitol 2015; 101(1): 57–63. doi: 10.1645/14-650.1 25357228

[25] Roche K., Pacciani E., Bianucci R., and Le Bailly M. Assessing the Parasitic Burden in a Late Antique Florentine Emergency Burial Site. Korean J Parasitol, 2019; 57(6), 587–593 doi: 10.3347/kjp.2019.57.6.587 31914509 PMC6960238

[26] Flammer P. G., Ryan H., Preston S. G., Warren S., Prichystalova R., Weiss R., et al. Epidemiological insights from a large-scale investigation of intestinal helminths in Medieval Europe. PLoS Negl Trop Dis 2020; 14(8): e0008600. doi: 10.1371/journal.pntd.0008600 32853225 PMC7451528

[27] Ledger M. L., Micarelli I., Ward D., Prowse T. L., Carroll M., Killgrove K., et al. Gastrointestinal infection in Italy during the Roman Imperial and Longobard periods: A paleoparasitological analysis of sediment from skeletal remains and sewer drains. Int J Paleopath, 2021; 33, 61–71. doi: 10.1016/j.ijpp.2021.03.001 33744834

[28] Brown L. D., Cai T. T., DasGupta A., Agresti A., Coull B. A., Casella G., et al. Interval estimation for a binomial proportion—Comment—Rejoinder. Statistical Science 2001; 16(2): 101–133.

[29] Team, R. *RStudio*: *Integrated Development for R*. 2015; RStudio Inc., Boston, MA.

[30] Team, R. C. R: A language and environment for statistical computing. 2017; R Foundation for Statistical Computing, Vienna, Austria.

[31] White T. D. and Folkens P. A. The human bone manual. Burlington, Mass. London, Elsevier 2005

[32] Bundy D. A. P., Kan S. P. and Rose R. Age-Related Prevalence, Intensity and Frequency-Distribution of Gastrointestinal Helminth Infection in Urban Slum Children from Kuala-Lumpur, Malaysia. Trans Roy Soc Trop Med Hyg 1988; 82(2): 289–294. doi: 10.1016/0035-9203(88)90450-6 3188158

[33] Bundy DAP, Appleby LJ, Bradley M, Croke K, Hollingsworth TD, Pullan R, et al., 100 Years of Mass Deworming Programmes: A Policy Perspective From the World Bank’s Disease Control Priorities Analyses. Adv Parasitol, 2018; 100: p. 127–154. doi: 10.1016/bs.apar.2018.03.005 29753337

[34] Wright J.E., Werkman M., Dunn J.C. and Anderson R. M. Current epidemiological evidence for predisposition to high or low intensity human helminth infection: a systematic review. *Parasites Vectors* 2018; 11, 65. doi: 10.1186/s13071-018-2656-4 29382360 PMC5791198

[35] Leles D., Reinhard K.J., Fugassa M., Ferriera L.F., Ineguez A.M., Araugo A. A parasitological paradox: Why is ascarid infection so rare in the prehistoric Americas? J Arch Sci, 2010; 37(7): 1510–1520.

[36] Bouchet F., and Le Bailly M. The Findings in Europe. In: Ferreira L.F., Reinhard K.J., and Araugo A., ed. Foundations of Paleoparasitology [online]. Rio de Janeiro: Editora FIOCRUZ, 2014, pp. 363–388. ISBN: 978-85-7541-598-6.

[37] Rácz S.E., De Araújo E.P., Jensen E., Mostek C., Morrow J.J., Van Hove M.L., et al. Parasitology in an archaeological context: analysis of medieval burials in Nivelles, Belgium. J Arch Sci, 2015; 53: 304–315

[38] Cote N.M. and Le Bailly M. Palaeoparasitology and palaeogenetics: review and perspectives for the study of ancient human parasites. Parasitology 2017; 145(5):656–664 doi: 10.1017/S003118201700141X 28747239

[39] Reinhard K. Reestablishing rigor in archaeological parasitology. Int J Paleopath, 2017; 19: 124–134. doi: 10.1016/j.ijpp.2017.06.002 29198394

[40] Camacho M., Araugo A., Morrow J., Buikstra J., Reinhard K. Recovering parasites from mummies and coprolites: an epidemiological approach. Parasit Vectors, 2018; 11(1): 248 doi: 10.1186/s13071-018-2729-4 29661215 PMC5902992

[41] Pike A. W. Recovery of helminth eggs from archaeological excavations, and their possible usefulness in providing evidence for the purpose of an occupation. Nature 1968; 219(5151): 303–304. doi: 10.1038/219303a0 5691459

[42] Araujo A., Reinhard K. J., Ferreira L. F. and Gardner S. L. Parasites as probes for prehistoric human migrations? Trends Parasitol 2008; 24(3): 112–115. doi: 10.1016/j.pt.2007.11.007 18262843

[43] Morand S. Phylogeography helps with investigating the building of human parasite communities. Parasitology 2012; 139(14): 1966–1974. doi: 10.1017/S0031182012000662 22717079

[44] Bouchet F., Guidon N., Dittmar K., Harter S., Ferreira L. F., Chaves S. M., et al. Parasite remains in archaeological sites. *Memorias do Instituto Oswaldo Cruz*, 2003; 98 Suppl 1, 47–52. doi: 10.1590/s0074-02762003000900009 12687762

[45] Jones A. K. G. and Nicholson C. Recent finds of *Trichuris* and *Ascaris* ova from Britain. Paleopathol Newsl 1988; 62: 5–6. 11621522

[46] McKenna W. J. B., Hutchinson A. R. and Jones A. K. G. Parasitological investigations on samples of sediment from excavations at 7–9 Aldwark, York. Ancient Monuments Laboratory Report Historic England 1988; 37/88.

[47] Kenward H. K., Dainton M., Kemenes I. and Carrott J. Environmental Evidence from insect remains and parasite eggs from the Lewthwaites Lane A Site, The Lanes, Carlisle. Ancient Monuments Laboratory Report, Historic England 1992; 77/92.

[48] Dark P. New evidence for the antiquity of the intestinal parasite Trichuris (whipworm) in Europe. Antiquity 2004; 78(301):676–681

[49] Ledger M.L., Rowan E., Marques F.G., Sigmier J.H., Šarkić N., Redžić S., et al. Intestinal parasitic infection in the eastern Roman Empire during the Imperial Period and Late Antiquity. American Journal of Archaeology, 2020;124(4), 631–657.

[50] Mitchell P. D. Human parasites in the Roman World: health consequences of conquering an empire. Parasitology 2017; 144(1): 48–58. doi: 10.1017/S0031182015001651 26741568

[51] De Rouffignac C. Parasite egg survival and identification from Hibernia Wharf, Southwark. London Archaeologist 1985; 5(4) 103: 105.

[52] Jones A. K. G. and Hutchinson A. R. Parasitological investigations on samples of organic sediment from excavations at Castle Street, Carlisle, Cumbria. Ancient Monuments Laboratory Report, Historic England 1988; 59.

[53] Jones A. K. G. and Maytom J. Parasitological investigations of the east annexe ditch. In Breeze D. J. ed. Bearsden: A Roman Fort on the Antonine Wall. Edinburgh, Society of Antiquaries of Scotland. 2016

[54] Jones A. K. G. Parasite ova from Roman levels at two sites within the Fortress of Eboracum: Two sites from the Bedren area of York. Ancient Monuments Laboratory Report. Historic England, 1984; 37

[55] Jones A. K. G. Parasitological investigations on samples of organic material associated with human burials at the Roman inhuman cemetery at Pondbury, Dorset. Ancient Monuments Laboratory Report. Historic England 1987; 40.

[56] Knights B. A., Dickson C. A., Dickson J. H. and Breeze D. J. Evidence concerning the Roman military diet at Bearsden, Scotland, in the 2nd Century AD. J Arch Sci 1983; 10(2): 139–152.

[57] Wilson A. and Rackham D. J. The environmental evidence from the Church Street Roman sewer system. In Addyman P. V. and Buckland P. C. The Archaeology of York. York Archaeological Trust. 1976; 14: 32–33.

[58] Carrott J., Issitt M., Kenward H., Lancaster S., Large F., Milles A. and Nicholson C. Assessment of insect remains, molluscs and parasite eggs from four sites in Lincoln (Site codes WF89, WN87, WNW88, WO89). Reports from the Environmental Archaeology Unit, York 1994; 94(12): 20.

[59] Carrott J., Dobney K., Hall A., Issitt M., Jaques D., Kenward H., et al. Assessment of biological remains from excavations at 22 Piccadilly (ABC Cinema), York (YAT/Yorkshire Museum sitecode 1987.21). Reports from the Environmental Archaeology Unit, York 1995; 95(5)3: 59.

[60] McKenna W. J. B., Hutchinson A. R., Jones A. K. G. and Nicholson C. Parasitological investigations on samples of organic sediment from excavations at Rougier Street, York. Ancient Monuments Laboratory Report, Historic England 1987; 227.

[61] Greig J. The Investigation of a Medieval Barrel-Latrine from Worcester. J Arch Sci 1981; 8(3): 265–282.

[62] Pike A. W. and Biddle M. Parasite Eggs in Medieval Winchester. Antiquity 1966; 40(160): 293–296.

[63] Taylor E. L. Parasitic helminths in mediaeval remains. Vet Record 1955; 67(12): 216–218.

[64] Tibesky K. and Sidell J. The parasite remains. In Malcolm G., Bowsher D. and Cowie R. eds. Middle Saxon London: excavations at the Royal Opera House, 1989–99. London, Museum of London Archaeology 2003: 333–337.

[65] Buckland P. C., Holdsworth P. and Monk M. Interpretation of a Group of Saxon Pits in Southampton. J. Arch Sci 1976; 3(1): 61–69.

[66] Pike A. W. Parasite eggs: the organic content of cesspit soil from Southampton, and their significance for the archaeologist and biologist. In Platt C. and Coleman-Smith R. eds. Excavations in Medieval Southampton 1953–1969. Leicester, Leicester University Press. 1975

[67] Jaques D., Schmidl A., Carrott J. and Beacock A. Biological Remains. In Hunter-Mann K. ed. Block E: Hungate Development York—A report on an archaeological excavation. York, York Archaeological Trust. 2008

[68] Akeret O., Carrott J., Mant J., Jaques D. and Gardner S. Environmental Samples. In B. Reeves 62–68 Low Petergate York, Assessment Report on an archaeological excavation. York, York Archaeological Trust. 2006

[69] Palliser D. M. The Cambridge Urban History of Britain: Volume 1: 600–1540. Cambridge, Cambridge University Press. 2000.

[70] Ferguson D. T. Nightsoil and the ’Great Divergence’: human waste, the urban economy, and economic productivity, 1500–1900. J Global History 2014; 9(3): 379–402.

[71] Oram R. D. Waste management and peri-urban agriculture in the early modern Scottish burgh. Agricultural History Review 2011; 59: 1–17.

[72] Jones A. K. G. and Phipps J. Entomological and parasitological investigations on samples from Christchurch, Spitalfields, London. Ancient Monuments Laboratory Report, Historic England 1988; 60.

[73] Anastasiou E., Mitchell P. D. and Jeffries N. The Paleoparasitology of 17th-18th century Spitalfields in London. In Mitchell P. D. and Buckberry J. editors. Proceedings of the 12th Conference of the British Association for Biological Anthropology and Osteoarchaeology. 2012; Oxford, BAR publishing: 53–61.

[74] Loe L., Webb H., Simmonds A and Poore D. The Patients’ Story: Dr Radcliffe’s Legacy in the Age of Hospitals. Excavations at the 18th - 19th century Radcliffe Infirmary Burial Ground. Oxford Archaeology Monographs (in press 2022)

[75] Jeffries N., Braybrooke T., Pearce J. and Wardle A. Development of the former Radcliffe Infirmary, Oxford, 1770–1900. Post-Medieval Archaeology 2015; 49(2): 238–268.

[76] Davenport R. J., Satchell M. and Shaw-Taylor L. M. W. Cholera as a "sanitary test’ of British cities, 1831–1866. History of the Family, 2019; 24(2): 404–438. 10.1080/1081602X.2018.1525755 PMC6582458 31274973

[77] Durey M. The return of the plague: British society and the cholera, 1831–2. Dublin; London, Gill and Macmillan. 1979

[78] Royal Commission 1845. The Second Report of the commissioners for inquiring into the state of large towns and populous districts. London, Clowes and Sons, p.87 Available from nationalarchives.gov.uk

[79] Crabtree P. J. Early Medieval Britain: The Rebirth of Towns in the Post-Roman West. Cambridge, Cambridge University Press. 2018

[80] Snow J. On the mode of communication of cholera. John Churchill Press, Princes Street, Soho London 1855

[81] Tynan N. Nineteenth century London water supply: Processes of innovation and improvement. The Review of Austrian Economics 2013; 26(1): 73–91.

[82] Hamlin C. Muddling in Bumbledom: on the enormity of large sanitary improvements in four British towns, 1855–1885. Victorian Studies 1988; 32(1): 55–83. 11623047

[83] Chatzakis M. K., Lyrintzis A. G., Mara D. D. and Angelakis A. N. Sedimentation Tanks through the Ages. Proceedings of the 1st IWA International Symposium on Water and Wastewater Technologies in Ancient Civilizations: 2006; 755–761.

[84] Maya C., Ortiz M. and Jimenez B. Viability of Ascaris and other helminth genera non larval eggs in different conditions of temperature, lime (pH) and humidity. Water Sci Technol 2010; 62(11): 2616–2624. doi: 10.2166/wst.2010.535 21099049

[85] Senecal J., Nordin A., & Vinnerås B. Fate of Ascaris at various pH, temperature and moisture levels. J of water and health, 2020; 18(3), 375–382. doi: 10.2166/wh.2020.264 32589622

[86] Stoll NR, This wormy world. J Parasitol, 1947; 33(1): p. 1–18. 20284977

[87] Boycott A. E. Further observations on the Diagnosis of *Ankylostoma* Infection with special reference to the Examination of the Blood. J Hyg (Lond) 1904; 4(4): 437–479. 10.1017/s0022172400002230 PMC2236037 20474200

[88] French H. S. and Boycott A. E. The Prevalence of *Trichocephalus dispar*. J Hyg (Lond) 1905; 5(3): 274–279 doi: 10.1017/s0022172400002540 20474224 PMC2236139

[89] Lynch D. M., Green E. A., McFadzean J. A. and Pugh I. M. *Trichuris trichiura* Infestations in the United Kingdom and Treatment with Difetarsone. Brit Med J. 1972; 4(5832): 73–76 doi: 10.1136/bmj.4.5832.73 5077469 PMC1786249

[90] Brooker S., Clements A. C. A. and Bundy D. A. P. Global epidemiology, ecology and control of soil-transmitted helminth infections. Adv Parasitol, 2006; 62: 221–261. doi: 10.1016/S0065-308X(05)62007-6 16647972 PMC1976253

[91] Trigg H. B., Jacobucci S. A., Mrozowski S. A and Steinberg J. M. Archaeological Parasites as Indicators of Environmental Change in Urbanizing Landscapes: Implications for Health and Social Status. American Antiquity 2017; 82(3): 517–535.

[92] Jones A. K. G. *Coprolites and faecal concretions*. In Bell M. Brean Down: Excavations 1983–1987. London, English Heritage: 1990; 242–245.

[93] Šebela L., Vojtková L. and Vojtek J. Intestinal parasites in man of old Bronze age. Anthropologie 1990; 28(1): 105–107.

[94] Wong M. S. and Bundy D. A. Quantitative assessment of contamination of soil by the eggs of *Ascaris lumbricoides* and *Trichuris trichiura*. Trans R Soc Trop Med Hyg 1990; 84(4): 567–570. doi: 10.1016/0035-9203(90)90043-e 2091353

