# Supplementary material for: Reconstructing the history of helminth prevalence in the UK
Source: PLoS Negl Trop Dis. 2022 Apr 21;16(4):e0010312. doi: 10.1371/journal.pntd.0010312 (PMC9022885; doi:10.1371/journal.pntd.0010312)
Supplement: Attachment — Submitted filename: Response to reviewers comments final.docx [file pntd.0010312.s001.docx]

Response to Reviewer comments: (Our responses in blue text)

Reviewer 1:

1. In which country/countries? **Response:** These infections have been detected in many countries including those in Europe and North America. We have added this indicator to the first sentence and some additional references.
2. Page 6: I think the paragraph on eradication should be deleted….” **Response:** We have deleted this paragraph.
3. Page 9 last paragraph (first paragraph of the results): this paragraph should be deleted as it summarized M&M and introduction. **Response:** We have deleted the “repetitive” middle section of this paragraph. We have left an introductory sentence to contextualise results and kept the results that have not been dealt with elsewhere.
4. The discussion is a bit long can it be condensed. **Response:** We have looked closely at the discussion and made some changes to reduce the length whilst addressing other requests. We hope we have achieved an appropriate balance.
5. Are there any study limitations, **Response:** We have added a short section to the discussion to mention the potential limitation of under-detection of eggs from archaeological material. Most helminth eggs are robust and are readily detected in samples from archaeological contexts and we did detect quite high prevalence rates. We have also commented upon the fact that burial-derived remains may not completely represent the structure of the living population.
6. Page 14 last paragraph and page 15: the comparison of the Czech site and the last paragraph should be deleted or moved as this already part of the discussion. **Response:** The paragraphs considering the influence of Pohansko in the medieval comparisons of UK and European sites have been condensed. We feel it is important to present some form of comparison in the results as the analysis was part of the current work.
7. It might be worth mentioning that hookworms rapidly hatch and therefore cannot be diagnosed unless samples are frozen or conserved, hence there might have been hookworm infections as well. **Response:** This is a good point but in the interests of brevity we didn’t feel it relevant to enter into a discussion of helminths that were not detected as we would have then had to include comments on others including *Enterobius vermicularis* which is surprisingly uncommon in archaeological contexts perhaps because of fragile eggs.
8. Last sentence (reviewer suggests removal as we already know what needs to be done). Response: We would like to retain part of the sentence as an indicator of how archaeological studies of disease might provide evidence for modern efforts. It simply tries to contextualise the benefit of studying past changes in disease patterns.

Reviewer 2: Materials and Methods

1. The sampling process could be more precise. Information should be added on where exactly samples from the pelvis were taken, i.e., above or below the pelvis. **Response:** Our apologies, we have added clarification that the samples originate from the ventral face of the sacrum (page 7).
2. The last sentence page 8 is written in a different police size. **Response:** We have corrected the font size.
3. Detail lacking in processing steps (this also comes up later in the review). **Response:** We apologise and have added more detail. We hope this is sufficient and provide a rationale as follows:

The reviewer is correct that there are a number of different protocols used to study parasites in archaeological contexts. Many involve a rehydration step either in water or 0.5% trisodium phosphate with some involving the use of other treatments such as acids or alkalis especially where mineralisation is an issue (as it can be with some coprolites). Some methods involve filtration to remove large debris and others involve density gradient based efforts at enrichment. Our approach was simple, rehydration with ultrapure water (none of the samples were mineralised) overnight with gentle agitation. We used water rather than 0.5% trisodium phosphate which is quite alkaline as we wished to avoid damage to eggs and ancient DNA (some of our samples are used for DNA analyses). Similarly we avoided the use of formaldehyde as a preservative due to its negative effects on DNA. Some methods use mechanical disruption with a mortar and pestle but this may also damage parasite eggs so we avoided this approach. Density gradient protocols may be good at enrichment of intact eggs but will be selective and less appropriate for any eggs with altered density (these may be recognisable but damaged). After rehydration we did remove large debris using filtration with coarse disposable nylon filters. Some methods use filtration with very small pore size filters (e.g. 20um) and then recover eggs from the filter which we considered likely to result in inefficient or variable recovery (we have not tested this).

The last point raised by the reviewer in this area was that of should we do more microscopy. Greater sensitivity might be achieved by performing more microscopy (some papers report 20 or more slides/sample), this would have been unachievable with hundreds of samples. However, our approach can identify levels of ~80 eggs/gram so should be sufficient to detect even quite light infections with adult worms (as considered by WHO (1987). We have made a comment in the methods section and mentioned it as one of the potential limitations in the discussion. It might have been important to perform more microscopy if we were calculating egg number/sample however we considered that this would be inappropriate with most burial associated samples containing low concentrations of eggs and variation due to factors outside our control (e.g. taphological events). Our methodology is simple, based upon rehydration of 5-7g of soil in water with gentle agitation before processing and direct examination of a set amount of the sample by microscopy. Our results are also presented as samples containing parasite eggs versus those where eggs were not detected. . The last point is that all samples in this study (and those in Flammer et al., 2020) employed the same method and are comparable.

It is clear that more comparisons of technical approach are appropriate (as presented by Dufour and LeBailly 2013) and these need to be performed on a variety of samples types including pelvic/sacral soil, coprolites (mineralised to different degrees) and communal deposits of different types. However, this is beyond the scope of the current manuscript. Interestingly, a recent paper used the trisodium rehydration/filter/pestle and mortar method and found similar rates of Ascaris in pelvic derived soils as we report (Roche et al., 2019).

Results

Reviewer 2

1. The first lines of this paragraph are repetitive. **Response:** We have removed the sentences that are repetitive with earlier sections.
2. Page 11, the use of the **acronym STH isn’t explained.**. **Response:** Thankyou, we have corrected this on first usage of the acronym.
3. Page 13: authors decide to divide individuals into two age-groups, but here again, this choice isn’t explained. Because of the collection, this appears to be motivated by the low number of individuals for some age class, having consequences on the statistic treatment. But this has to be state. **Response:** Our rationale was twofold, first in modern endemic areas school age children are known to be more affected than adults (Bundy et al., 1988; 2018; Wright et al., 2018). We didn’t divide the groups to a finer scale as this would have excluded samples and made group sizes too small for robust statistical analysis. We have clarified this in the text.
4. Page 15, line 7, Trichuris has to be italicized. **Response:** We have corrected and checked for other parasites too.
5. Page 18, author states that previous data in England were unsuitable for calculating prevalence. Why? It is not possible to consider the number of positive samples from a site on which latrine are analyzed? **Response:** We have clarified this in the text. Prevalence (the number of affected individuals) can not be determined from communal deposits (with an unknown number of contributors). A few single individual analyses exist but the numbers examined were too small to gain accurate measures of prevalence. We had explained this in the discussion but have added a short text at the position indicated by the reviewer.

Reviewer #3: 1. The results presented were clear, precise and well-described.
2. The figures (tables, images) were clear. Legends were detailed and self-explanatory.
**Response:** No requested changes.

Discussion:

Reviewer #2:

1. The reviewer makes a range of points and requests more citations of European work. **Response**: The manuscript is focussed on English sites hence the bias towards citations relevant to the UK. We did cite a number of papers that deal with European circumstances and have added more. There are very few papers that analyse sufficient single individuals in any context to allow for analysis of prevalence or frequency of infection in individuals. This limited the relevance of many publications to anything other than broad comments.
2. A sentence page 20, lines 13-14, justify an observation of the results from the Industrial period, on the low rate of Trichuris, referring to Akeret et al 2006. It refers to samples prepared using palynological techniques, including acids and bases for preparation, known as destroying parasite eggs, except Ascaris which are more resistant. Response: We agree that harsh treatments can affect the detection of parasite eggs. However, we have carefully re-examined the Akaret paper and the parasite analyses employ a simple semi-quantitative squash method with water rehydration (referencing the method discussed by Dainton 1992) hence does not use harsh treatments. The report mentions both Ascaris and Trichuris with greater numbers identified in samples that date from medieval periods compared with the single Trichuris egg identified in an Industrial period. We therefore retain the sentence but clarify the presence of Ascaris and Trichuris rather than stating STH for the earlier samples.

Reviewer #3: The authors have done an excellent job of combining history/archaeology with parasitic epidemiology. The entire paper was a delight to read. However I have 2 points of concern :- 1. I believe the references listed in the paper may not be the correct way to present. 2. Please add line numbers to the manuscript, so that it's easier to review. **Response**. Thankyou we have made both changes.

Editorial/Data presentation

Reviewer #2:

1. Bibliography. **Response** Thankyou. We apologise for these errors and have checked the references.
2. The photo of the Trichuris egg isn't in a good quality. **Response:** We consider the photomicrograph of Trichuris reasonably good and it is possible to see one of the polar plugs very clearly as well as material within the egg. Many of the eggs observed were lacking polar plugs (as is often the case with archaeological samples) which is why we selected this image.
3. In the all text, the author should use the new accepted name for the fish tapeworm which is no more Diphyllobothrium latum, but as stated page 4, Dibothriocephalus latus. **Response:** As we indicated on page 4 these names are synonyms and whilst we support much of the rationale given in Waeschenbach et al., 2017 for reviving the Dibothriocephalus genus there are still some question that remain (beyond the scope of the current work). The “new name” is not yet firmly established in the literature and Pubmed searches identify recent papers using either name. We used D. latum since this has been most widely used for many years but reported its synonym. We have also added the synonym to the abstract to facilitate online searches. If the editor feels strongly we will change the naming of this parasite.
4. Bouchet 2003 should be added to the first sentence of the introduction. Response: We have added Bouchet et al. 2003
5. Other references for Europe: Response, We apologise for not including some of these in the final submitted manuscript (some appear to have been lost during our revisions) and have added some to relevant parts of the introduction and discussion. The focus of the paper was on English sites and the European comparisons are relevant in some places.
6. Assessment of prevalence. The statement of the authors (page 5) writing that “prevalence rates can be determined” based on the egg recovery on skeleton isn’t true, when considering the real definition of the “prevalence” in epidemiology. When sampling on skeletons, the sample do not only contain ancient fecal matter, but fecal matter mixed with an unknown amount of earth/soil, which block the calculation of the prevalence. The only case I know from archaeology on which such calculation was pertinent is in Racz et al 2015 (JAS). The sentence and the following work are valid only if the authors explain their definition of the “prevalence”. This explanation arrives too late page 6 and should be mentioned earlier.
    Response: We respectfully disagree with this statement. Prevalence is defined as the proportion of a population with a disease or a particular condition. It is of course affected by the sensitivity of detection methods but reporting the proportion of a population where eggs are detected is a measure of prevalence. We carefully defined what was being presented as prevalence at the appropriate part of the introduction (we did look to move it earlier in the introduction [as suggested] but this would not be possible without adding a paragraph of text). Reviewer 1 asked if there were any study limitations and in response to this we have added a short statement to the discussion. We hope this is sufficient. The study by Racz et al., 2015 is an excellent example of enumerating parasite egg numbers in coprolites obtained from three burials. There are lots of positive elements to this analysis (e.g. potential variation in parasite load) but examining three individuals is not a robust way of determining the proportion of affected individuals in a population.

References

Bundy, D. A. P., S. P. Kan and R. Rose. *Age-Related Prevalence, Intensity and Frequency-Distribution of Gastrointestinal Helminth Infection in Urban Slum Children from Kuala-Lumpur, Malaysia.* Trans Roy Soc Trop Med Hyg 1988; **82**(2): 289-294.

Bundy DAP, Appleby LJ, Bradley M, Croke K, Hollingsworth TD, Pullan R, et al., 100 Years of Mass Deworming Programmes: A Policy Perspective From the World Bank’s Disease Control Priorities Analyses. Adv Parasitol, 2018. 100: p. 127–154. https://doi.org/10.1016/bs.apar.2018.03.005 PMID: 29753337

Dainton, M., 1992. ‘A quick, semi quantitative method for recording nematode gut parasite eggs from archaeological deposits’, Circaea 9, 58-63

Dufour, B., & Le Bailly, M. (2013). Testing new parasite egg extraction methods in paleoparasitology and an attempt at quantification. *International journal of paleopathology*, *3*(3), 199–203.

Flammer, P. G., H. Ryan, S. G. Preston, S. Warren, R. Prichystalova, R. Weiss, V. Palmowski, S. Boschert, K. Fellgiebel, I. Jasch-Boley, M. S. Kairies, E. Rummele, D. Rieger, B. Schmid, B. Reeves, R. Nicholson, L. Loe, C. Guy, T. Waldron, J. Machacek, J. Wahl, M. Pollard, G. Larson and A. L. Smith. *Epidemiological insights from a large-scale investigation of intestinal helminths in Medieval Europe.* PLoS Negl Trop Dis 2020; 14(8): e0008600

Rácz, S.E., De Araújo, E.P., Jensen, E., Mostek, C., Morrow, J.J., Van Hove, M.L., Bianucci, R., Willems, D., Heller, F., Araújo, A. and Reinhard, K.J., 2015. Parasitology in an archaeological context: analysis of medieval burials in Nivelles, Belgium. *Journal of Archaeological Science*, *53*, pp.304-315.

Roche, K., Pacciani, E., Bianucci, R., & Le Bailly, M. (2019). Assessing the Parasitic Burden in a Late Antique Florentine Emergency Burial Site. *The Korean journal of parasitology*, *57*(6), 587–593.

Waeschenbach, A., Brabec, J., Scholz, T., Littlewood, D., & Kuchta, R. (2017). The catholic taste of broad tapeworms - multiple routes to human infection. *International journal for parasitology*, *47*(13), 831–843.

World Health Organization. (1997) *Prevention and control of intestinal parasitic infections: report of a WHO expert committee*

Wright, J.E., Werkman, M., Dunn, J.C. *et al.* Current epidemiological evidence for predisposition to high or low intensity human helminth infection: a systematic review. *Parasites Vectors* **11,** 65 (2018).
