# Supplementary material for: Reconstructing the history of helminth prevalence in the UK
Source: PLoS Negl Trop Dis. 2022 Apr 21;16(4):e0010312. doi: 10.1371/journal.pntd.0010312 (PMC9022885; doi:10.1371/journal.pntd.0010312)
Supplement: Peer review history

# Peer review: Decision Letter 0

**DOI:** 10.1371/journal.pntd.0010312.r001

**Authors:** Subash Babu, Maria Victoria Periago

23 Dec 2021

Dear Prof Smith,

Thank you very much for submitting your manuscript "Reconstructing the history of helminth prevalence in the UK" for consideration at PLOS Neglected Tropical Diseases. As with all papers reviewed by the journal, your manuscript was reviewed by members of the editorial board and by several independent reviewers. In light of the reviews (below this email), we would like to invite the resubmission of a significantly-revised version that takes into account the reviewers' comments. 

  

We cannot make any decision about publication until we have seen the revised manuscript and your response to the reviewers' comments. Your revised manuscript is also likely to be sent to reviewers for further evaluation.

When you are ready to resubmit, please upload the following:

[1] A letter containing a detailed list of your responses to the review comments and a description of the changes you have made in the manuscript. Please note while forming your response, if your article is accepted, you may have the opportunity to make the peer review history publicly available. The record will include editor decision letters (with reviews) and your responses to reviewer comments. If eligible, we will contact you to opt in or out.

[2] Two versions of the revised manuscript: one with either highlights or tracked changes denoting where the text has been changed; the other a clean version (uploaded as the manuscript file). 

Important additional instructions are given below your reviewer comments.

Please prepare and submit your revised manuscript within 60 days. If you anticipate any delay, please let us know the expected resubmission date by replying to this email. Please note that revised manuscripts received after the 60-day due date may require evaluation and peer review similar to newly submitted manuscripts.

Thank you again for your submission. We hope that our editorial process has been constructive so far, and we welcome your feedback at any time. Please don't hesitate to contact us if you have any questions or comments.

Sincerely,

Subash Babu

Associate Editor

PLOS Neglected Tropical Diseases

Maria Periago

Deputy Editor

PLOS Neglected Tropical Diseases

***********************

Reviewer's Responses to Questions

**Key Review Criteria Required for Acceptance?**

As you describe the new analyses required for acceptance, please consider the following:

**Methods**

-Are the objectives of the study clearly articulated with a clear testable hypothesis stated?

-Is the study design appropriate to address the stated objectives?

-Is the population clearly described and appropriate for the hypothesis being tested?

-Is the sample size sufficient to ensure adequate power to address the hypothesis being tested?

-Were correct statistical analysis used to support conclusions?

-Are there concerns about ethical or regulatory requirements being met?

Reviewer #1: This is a well written and interesting manuscript and I have only few comments. 

Introduction

First line: in which country/countries?

Page 6: I think the paragraph on eradication should be deleted as it is a bit out of focus. Moreover, it is elimination (as a public health problem) and not eradication 

Results 

Page 9 last paragraph (first paragraph of the results): this paragraph should be deleted as it summarized M&M and introduction. 

Page 14 last paragraph and page 15: the comparison of the Czech site and the last paragraph should be deleted or moved as this already part of the discussion

Discussion

The discussion is a bit long, hence I would suggest condensing it a bit. 

Are there any study limitations?

It might be worth mentioning that hookworms rapidly hatch and therefore cannot be diagnosed unless samples are frozen or conserved, hence there might have been hookworm infections as well. 

Last sentence: Understanding the factors that may have affected the past prevalence of STH infections may provide key information for modern control efforts, particularly where anthelminthic programmes have struggled to provide a long-lasting solution. 

I would delete this sentence: we know what is needed to control these infections but implementation is often challenging

Reviewer #2: Material & method

The sampling process could be more precise. Information should be added on where exactly samples from the pelvis were taken, i.e., above or below the pelvis.

The last sentence page 8 is written in a different police size. 

In the processing and microscopy section: why authors decided to use a rehydration method different to all what is readable in the bibliography dealing with ancient parasites? The rehydration is performed with ultrapure water. No screening steps. “Two rounds of microscopy” which isn’t clear according to me. Does it mean two slides? 

The rehydration techniques used in the different groups working and developing paleoparasitology or archaeoparasitology, even if not exactly the same, consist in more or less 3 steps:

The rehydration, generally in TSP, which help in disaggregation of the sample and the parasite egg suspension.

The screening step, discarding the bigger elements which present no interest and can be problematic during microscopic observation.

The sedimentation.

The choice of the authors has to be explained.

Reviewer #3: 1. The objectives of the study were clearly articulated, well-written and easy to understand. 

2. The study was designed in detail. 

3. The population was clearly described and appropriate. 

4. The sample size was sufficient to support the conclusions. 

5. I did not find any concerns regarding ethical or regulatory requirements not being met.

--------------------

**Results**

-Does the analysis presented match the analysis plan?

-Are the results clearly and completely presented?

-Are the figures (Tables, Images) of sufficient quality for clarity?

Reviewer #1: (No Response)

Reviewer #2: Results:

The first lines of this paragraph were already written in the previous section. This should be shortened or deleted.

Page 11, the use of the acronym STH isn’t explained before. Probably Soil Transmitted Helminths. But the entire terminology has to be used one time with the acronym to be clear and correct.

Page 13: authors decide to divide individuals into two age-groups, but here again, this choice isn’t explained. Because of the collection, this appears to be motivated by the low number of individuals for some age class, having consequences on the statistic treatment. But this has to be state.

As a consequence, page 14, it is mentioned that cestods (Taenia and Dibothriocephalus) were only detected in older individuals, older than 13 yo.

Page 15, line 7, Trichuris has to be italicized.

Reviewer #3: 1. The results presented were clear, precise and well-described. 

2. The figures (tables, images) were clear. Legends were detailed and self-explanatory.

--------------------

**Conclusions**

-Are the conclusions supported by the data presented?

-Are the limitations of analysis clearly described?

-Do the authors discuss how these data can be helpful to advance our understanding of the topic under study?

-Is public health relevance addressed?

Reviewer #1: (No Response)

Reviewer #2: Discussion:

My previous comment on the bibliography also concerned this part. This paper proposition concerns European archaeological sites and the authors cite 6 American papers on the 8 citations appearing in the first sentence…

Some affirmation in the discussion appears problematic according to me. Of course, they are directly linked to the results, and the previous results from Flammer et al 2020 (cited 16 times in the all text), but no discussion are made on the samples themselves neither the extraction method and observation.

I mean, working on skeleton remains never allowed to observe the maximum of parasite remains under microscopy. Because of taphonomical processes, including postmortem body preparation (exposition, washing, intestine removal…), and the intense microbiological activity, eggs disappear.

The extraction method, which isn’t conventional (very different from the one used in the other labs), also is a problem. The microscopical observation, if only two slide per sample were observed also is a problem.

This led to conclusion which are not true, and in opposition with previous observation from cesspit/latrine contexts for example. 

Page 18, author states that previous data in England were unsuitable for calculating prevalence. Why? It is not possible to consider the number of positive samples from a site on which latrine are analyzed?

Same page, line 8, Ascaris has to be italicized.

A sentence page 20, lines 13-14, justify an observation of the results from the Industrial period, on the low rate of Trichuris, referring to Akeret et al 2006. It refers to samples prepared using palynological techniques, including acids and bases for preparation, known as destroying parasite eggs, except Ascaris which are more resistant.

Reviewer #3: The authors have done an excellent job of combining history/archeology with parasitic epidemiology. The entire paper was a delight to read. However I have 2 points of concern :- 1. I believe the references listed in the paper may not be the correct way to present. 2. Please add line numbers to the manuscript, so that it's easier to review.

--------------------

**Editorial and Data Presentation Modifications?**

Use this section for editorial suggestions as well as relatively minor modifications of existing data that would enhance clarity. If the only modifications needed are minor and/or editorial, you may wish to recommend “Minor Revision” or “Accept”. 

Reviewer #1: (No Response)

Reviewer #2: Bibliography

The entire bibliography has to be check as many ref do not appear in the text :

Cox, 2002

Loreille et al 2001

Pullan & Brooker 2012

Soe et al 2015

Torok et al 2016.

Wrigley 1985.

The date in the ref Lynch et al 1985 has to be check as different in the text and the bibliography section.

Finally, some ref in the text are not cited in the bibliography:

Ledger et al 2020

Senecal et al 2020

The photo of the Trichuris egg isn't in a good quality. This has to be modified.

Reviewer #3: 1. I believe the references listed in the paper may not be the correct way to present. 

2. Please add line numbers to the manuscript, so that it's easier to review.

--------------------

**Summary and General Comments**

Use this section to provide overall comments, discuss strengths/weaknesses of the study, novelty, significance, general execution and scholarship. You may also include additional comments for the author, including concerns about dual publication, research ethics, or publication ethics. If requesting major revision, please articulate the new experiments that are needed.

Reviewer #1: (No Response)

Reviewer #2: General comments:

The article proposition deals with the analysis the variation of the percentage of positive samples (rate) after microscopic analysis of intestinal parasites in samples taken from human skeleton from archaeological sites in England.

This paper is well written and understandable.

However, many complements has to be integrate before being acceptable for publication.

Below the authors could find some comments and modification proposition for the text.

Abstract:

In the all text, the author should use the new accepted name for the fish tapeworm which is no more Diphyllobothrium latum, but as stated page 4, Dibothriocephalus latus.

Introduction:

From the beginning of the text, and later in the discussion, I am perplexed on the fact that large part of the European bibliography on ancient parasites is omitted. Bouchet, in France, published articles on the subject from 1989 to 2016, most part on European contexts, and is only cited one time. Herrmann, in Germany, published articles on cites dated to the medieval period in the late 1980’s. In a general comment, almost all the French work is forgotten. Dommelier, Le Bailly, Maicher, Dufour. All publishing in international journals.

Of course, English researchers as Jones, Pike, and Mitchell are well cited.

In the first introduction sentence, please add European references. Bouchet et al 2003 should be added.

The statement of the authors (page 5) writing that “prevalence rates can be determined” based on the egg recovery on skeleton isn’t true, when considering the real definition of the “prevalence” in epidemiology. When sampling on skeletons, the sample do not only contain ancient fecal matter, but fecal matter mixed with an unknown amount of earth/soil, which block the calculation of the prevalence.

The only case I know from archaeology on which such calculation was pertinent is in Racz et al 2015 (JAS).

The sentence and the following work are valid only if the authors explain their definition of the “prevalence”. This explanation arrives too late page 6 and should be mentioned earlier.

Reviewer #3: (No Response)

--------------------

PLOS authors have the option to publish the peer review history of their article (what does this mean?). If published, this will include your full peer review and any attached files.

If you choose “no”, your identity will remain anonymous but your review may still be made public.

**Do you want your identity to be public for this peer review?** For information about this choice, including consent withdrawal, please see our Privacy Policy.

Reviewer #1: No

Reviewer #2: No

Reviewer #3: No

Figure Files:

While revising your submission, please upload your figure files to the Preflight Analysis and Conversion Engine (PACE) digital diagnostic tool, https://pacev2.apexcovantage.com. PACE helps ensure that figures meet PLOS requirements. To use PACE, you must first register as a user. Then, login and navigate to the UPLOAD tab, where you will find detailed instructions on how to use the tool. If you encounter any issues or have any questions when using PACE, please email us at figures@plos.org.

Data Requirements:

Please note that, as a condition of publication, PLOS' data policy requires that you make available all data used to draw the conclusions outlined in your manuscript. Data must be deposited in an appropriate repository, included within the body of the manuscript, or uploaded as supporting information. This includes all numerical values that were used to generate graphs, histograms etc.. For an example see here: http://www.plosbiology.org/article/info%3Adoi%2F10.1371%2Fjournal.pbio.1001908#s5.

Reproducibility:

To enhance the reproducibility of your results, we recommend that you deposit your laboratory protocols in protocols.io, where a protocol can be assigned its own identifier (DOI) such that it can be cited independently in the future. Additionally, PLOS ONE offers an option to publish peer-reviewed clinical study protocols. Read more information on sharing protocols at https://plos.org/protocols?utm_medium=editorial-email&utm_source=authorletters&utm_campaign=protocols

# Author comment: Author response to Decision Letter 0

**DOI:** 10.1371/journal.pntd.0010312.r002

4 Feb 2022

Attachment Submitted filename: Response to reviewers comments final.docx Click here for additional data file.

# Peer review: Decision Letter 1

**DOI:** 10.1371/journal.pntd.0010312.r003

**Authors:** Subash Babu, Maria Victoria Periago

7 Mar 2022

Dear Prof Smith,

We are pleased to inform you that your manuscript 'Reconstructing the history of helminth prevalence in the UK' has been provisionally accepted for publication in PLOS Neglected Tropical Diseases.

Before your manuscript can be formally accepted you will need to complete some formatting changes, which you will receive in a follow up email. A member of our team will be in touch with a set of requests.

Please note that your manuscript will not be scheduled for publication until you have made the required changes, so a swift response is appreciated.

IMPORTANT: The editorial review process is now complete. PLOS will only permit corrections to spelling, formatting or significant scientific errors from this point onwards. Requests for major changes, or any which affect the scientific understanding of your work, will cause delays to the publication date of your manuscript.

Should you, your institution's press office or the journal office choose to press release your paper, you will automatically be opted out of early publication. We ask that you notify us now if you or your institution is planning to press release the article. All press must be co-ordinated with PLOS.

Thank you again for supporting Open Access publishing; we are looking forward to publishing your work in PLOS Neglected Tropical Diseases.

Best regards,

Subash Babu

Associate Editor

PLOS Neglected Tropical Diseases

Maria Periago

Deputy Editor

PLOS Neglected Tropical Diseases

***********************************************************

Reviewer's Responses to Questions

**Key Review Criteria Required for Acceptance?**

As you describe the new analyses required for acceptance, please consider the following:

**Methods**

-Are the objectives of the study clearly articulated with a clear testable hypothesis stated?

-Is the study design appropriate to address the stated objectives?

-Is the population clearly described and appropriate for the hypothesis being tested?

-Is the sample size sufficient to ensure adequate power to address the hypothesis being tested?

-Were correct statistical analysis used to support conclusions?

-Are there concerns about ethical or regulatory requirements being met?

Reviewer #1: (No Response)

Reviewer #2: All comments were considered and received were discussed in the author answer or added in the new version.

Reviewer #3: (No Response)

**********

**Results**

-Does the analysis presented match the analysis plan?

-Are the results clearly and completely presented?

-Are the figures (Tables, Images) of sufficient quality for clarity?

Reviewer #1: (No Response)

Reviewer #2: All comments were considered and received were discussed in the author answer or added in the new version.

Reviewer #3: (No Response)

**********

**Conclusions**

-Are the conclusions supported by the data presented?

-Are the limitations of analysis clearly described?

-Do the authors discuss how these data can be helpful to advance our understanding of the topic under study?

-Is public health relevance addressed?

Reviewer #1: (No Response)

Reviewer #2: All comments were considered and received were discussed in the author answer or added in the new version.

Reviewer #3: (No Response)

**********

**Editorial and Data Presentation Modifications?**

Use this section for editorial suggestions as well as relatively minor modifications of existing data that would enhance clarity. If the only modifications needed are minor and/or editorial, you may wish to recommend “Minor Revision” or “Accept”.

Reviewer #1: (No Response)

Reviewer #2: (No Response)

Reviewer #3: (No Response)

**********

**Summary and General Comments**

Use this section to provide overall comments, discuss strengths/weaknesses of the study, novelty, significance, general execution and scholarship. You may also include additional comments for the author, including concerns about dual publication, research ethics, or publication ethics. If requesting major revision, please articulate the new experiments that are needed.

Reviewer #1: The authors have addressed all comments

Reviewer #2: All comments were considered and received were discussed in the author answer or added in the new version.

Reviewer #3: (No Response)

**********

PLOS authors have the option to publish the peer review history of their article (what does this mean?). If published, this will include your full peer review and any attached files.

If you choose “no”, your identity will remain anonymous but your review may still be made public.

**Do you want your identity to be public for this peer review?** For information about this choice, including consent withdrawal, please see our Privacy Policy.

Reviewer #1: No

Reviewer #2: No

Reviewer #3: No

# Editor report: Acceptance letter

**DOI:** 10.1371/journal.pntd.0010312.r004

**Authors:** Subash Babu, Maria Victoria Periago

28 Mar 2022

Dear Prof Smith,

We are delighted to inform you that your manuscript, "Reconstructing the history of helminth prevalence in the UK," has been formally accepted for publication in PLOS Neglected Tropical Diseases.

We have now passed your article onto the PLOS Production Department who will complete the rest of the publication process. All authors will receive a confirmation email upon publication.

The corresponding author will soon be receiving a typeset proof for review, to ensure errors have not been introduced during production. Please review the PDF proof of your manuscript carefully, as this is the last chance to correct any scientific or type-setting errors. Please note that major changes, or those which affect the scientific understanding of the work, will likely cause delays to the publication date of your manuscript. Note: Proofs for Front Matter articles (Editorial, Viewpoint, Symposium, Review, etc...) are generated on a different schedule and may not be made available as quickly.

Soon after your final files are uploaded, the early version of your manuscript will be published online unless you opted out of this process. The date of the early version will be your article's publication date. The final article will be published to the same URL, and all versions of the paper will be accessible to readers.

Thank you again for supporting open-access publishing; we are looking forward to publishing your work in PLOS Neglected Tropical Diseases. 

Best regards,

Shaden Kamhawi

co-Editor-in-Chief

PLOS Neglected Tropical Diseases

Paul Brindley

co-Editor-in-Chief

PLOS Neglected Tropical Diseases
